# The experience of nurses caring for children with intellectual disability

**DOI:** 10.3389/fpsyt.2025.1688236

**Published:** 2025-11-18

**Authors:** Salmah Alghamdi, Rasha Alsaigh, Hawa Alabdulaziz, Nada Dahlawi, Afnan Gmmash

**Affiliations:** 1 Maternity and Child Nursing Department, Faculty of Nursing, King Abdulaziz University, Jeddah, Saudi Arabia; 2 Department of Physical Therapy, Faculty of Medical Rehabilitation Sciences, King Abdulaziz University, Jeddah, Saudi Arabia

**Keywords:** nurse, experience, children, intellectual disability, qualitative

## Abstract

**Introduction:**

Children with disabilities are a vulnerable population and may have frequent hospital admissions. Therefore, nurses should be prepared to provide comprehensive care for this group of children. The aim of this research is to explore the experience of nurses caring for children with intellectual disabilities (ID).

**Methods:**

A qualitative descriptive design was used. A sample of eight registered nurses was recruited from different pediatric clinical settings through social media. The inclusion criteria were that nurses had to be registered, had experience providing care for children with ID for at least one year, and could speak English or Arabic. The data were collected using semi-structured interviews. Thematic analysis was used for data analysis.

**Results:**

The study revealed four major themes: (1) Communication barriers and the need for caregivers/parents’ interpretation, (2) Increased nursing demands due to complex care, (3) Adaptation to the greater complexity of care, and (4) Need for support for both nurses and caregivers/parents.

**Conclusion:**

Findings from this study revealed that nurses are facing various challenges while committed to providing compassionate care for children with disabilities. Efforts should be made to provide support for families and nurses to ensure comprehensive care for children with ID.

## 1 Introduction

Intellectual disability (ID) has been defined as a condition of below-average intelligence or mental ability, with multiple severity levels (1). It is characterized by a significant impairment in both intellectual functioning and adaptive behavior, existing concurrently with related limitations in two or more areas of daily life functioning including communication and self-care (2, 3).

According to the Centers for Disease Control and Prevention (4), the prevalence of children diagnosed with ID in the United States was 1.48% among boys and 0.90% among girls during 2014–2016. There is a lack of recent statistics on the prevalence of ID among children. A study built on a population-based national survey estimated the prevalence of ID in Saudi Arabia as 8.9 per 1,000 children (5).

Children with disabilities are a vulnerable population who require specialized care. According to a study by Mimmo et al., children with ID accounted for over 14% of admissions to a tertiary pediatric healthcare facility (6). Compared to their peers, these children also experienced higher median admission costs, a significantly longer median length of stay, and more frequent admissions due to high rates of morbidity (6, 7). Negative health outcomes for these cohorts have been linked globally to nurses’ poor levels of confidence and educational readiness to provide care for them (8, 9). In general, planning and providing care for a child with an ID and other developmental comorbidities frequently presents additional difficulties (10, 11).

According to Trollor et al., many undergraduate medical and nursing curricula provide little exposure to the health of people with ID (12). The development of skills and a positive outlook when participating in effective communication, relationships based on mutual trust, and abilities in caregiving and behavior management are the foundations of staff empowerment (13, 14). Thus, it is highly advised that undergraduate and graduate health professions programs include more instruction on how to care for children with ID (12, 15). For the benefit of the patient, family, and staff, collaborating with parents enhances overall safety and care quality (16).

Nurses play major role in providing care for patients. The conceptual framework by Jean Watson’s Theory of Human Caring was used in this study to guide the understanding of the key concepts (17). A qualitative Australian study by Lewis et al. (18) revealed that nurses often experience a range of challenges when caring for children with ID, communication difficulties, and lack of knowledge or training. Cameron and Jones (19) also found that nurses experience a range of emotions when caring for children with disabilities including satisfaction and overwhelming.

Australia’s 2020 national survey of 693 registered nurses was the most important study examining the association among these important aspects: “education, confidence, comfort and preparedness” (20). Nurses who have received education related to caring for individuals with ID report feeling more comfortable, confident, and prepared (20). Moreover, Indonesian nurses’ self-perceived comfort, confidence, knowledge, and readiness to care for individuals with ID were insufficient, as were their educational experiences (21). More than half of the world’s health workforce is made up of nurses and midwives (22). However, studies on nurses’ perspectives on and experiences of providing high-quality care for children with ID who are admitted to hospitals on a regular basis are limited in the literature. There are lack of studies exploring the experience of nurses caring for children with ID in Saudi Arabia. Thus, this study aimed to explore the experience of nurses caring for children with ID.

## 2 Materials and methods

### 2.1 Study design and participants

This study was a descriptive qualitative research design to explore the experiences of nurses caring for children with disabilities in the clinical settings. This descriptive qualitative design was used to find an answer to “what,”- “why,”- and “how”-type questions regarding the experience of caring for children with ID in Saudi Arabia.

#### 2.1.1 Setting

Nurses were recruited from different pediatric clinical settings at hospitals and other health care centers via social media (WhatsApp and X). An invitation link was created using Google Forms that includes the consent of the study where participants can fill in their contact information if they are willing to participate in the study. The researcher then reached out to the participants to schedule the appointment for the interview.

#### 2.1.2 Sample

This study included a sample of registered nurses. Purposeful sampling technique was used to recruit nurses with previous experience in caring for children with ID. ID in this study is related to significant intellectual and adaptive deficits, with limitations in daily functions (2, 3). The inclusion criteria were registered nurses for at least 1 year, had experience in providing care for children with ID at least once in the previous 5 years, and spoke English or Arabic.

#### 2.1.3 Sample size

The recommended sample size for this study was between 5 and 25 participants as suggested for qualitative research by Creswell (23). After analyzing the last three interviews, there was no new information, themes, or concepts emerged from the incoming data. The research team agreed that the data were rich and comprehensive to address the research purposes, at this point we determined the saturation. The recruitment of new participants was discontinued.

#### 2.1.4 Data collection/instrumentation

The data were collected using semi-structured interviews and open-ended questions. The interview questions were developed based on a review of the relevant literature including the following questions: Tell me about your experience of providing nursing care to a child with intellectual disability (ID), Describe your experience in dealing with the family of a child with ID, How does caring for a child with ID impact on your daily workload, How do nurses manage the unique needs of children with ID, What are your recommendations to improve the quality of care provided for children with ID? The interviews were conducted in person or ZOOM by researchers in a private area. The interviews lasted approximately 30–60 minutes and were recorded. The data collection period spanned from February 2024 until reaching saturation in December 2024.

#### 2.1.5 Data analysis

Thematic analysis was used to analyze the data. The researchers analyzed the data as they collected them, searching for common patterns and themes. The following steps were taken for data analysis and guided by Braun and Clarke (2006) framework of thematic analysis (24). The analysis started with transcribing the audio recordings into texts. The researchers became familiar with the data by reading them, then generated initial codes to the text by identifying common key words and phrases. Initial coding was performed independently by at least two researchers (the authors). Each researcher read and coded the transcripts, and then we compared our coding. This process was followed by a collaborative review where both coders met to compare their coding logs and discuss discrepancies. Discrepancies were discussed until consensus was reached. In cases of disagreement, a third researcher was consulted. This was followed by merging the codes into themes. The themes were reviewed, and the final themes and subthemes were identified and presented in the results. The research team consisted of experienced, specialized pediatric nurses familiar with qualitative research and caring for children with disabilities. This background supported a deeper understanding of participants’ experiences. The trustworthiness of the analysis was ensured through codebook, and reflexivity. To ensure trustworthiness of findings, all authors aligned the data in the codebook separately, then met and discussed the results until a high percentage of agreement was reached. All authors participated in keeping a reflexive journal to track their feelings, assumptions, and progress in analysis. Furthermore, a member check was performed through sending to random participants their transcribed text and drafted thematic analysis for validation. The authors maintained a detailed audit of data collection and analysis processes.

### 2.2 Ethical consideration

Ethical approval was obtained from the faculty of nursing at King Abdulaziz University (Ref. No. 2F.38). In addition, ethical approval was obtained from King Abdulaziz University Hospital (Ref. No. 634-23). Informed consent was completed prior to the interview. Confidentiality of the participants and research data was maintained throughout the research process.

## 3 Results

### 3.1 Demographic characteristics

A total of eight female nurses were interviewed and included in this study. The sample included full-time nurses from different age groups. The majority of nurses were aged between 30 and 39 years old and married (n=5, 62.5%), had a bachelor’s degree (n=6, 75%), and had at least 5 years of experience (n=4, 50%). Detailed demographics characteristics are presented in **Table 1**.

**Table 1:** Demographic characteristics of the participants (N = 8).

Nurses’ Characteristics	Frequency (n)	Percentage %
Age		
Between 20 and 29	2	25
Between 30 and 39	5	62.5
Between 40 and 49	1	12.5
50 and above	0	0
Marital status		
Married	5	62.5
Single	2	25
Separated	1	12.5
Employment status		
Working part time	0	0
Working full time (at least 35 hours per week)	8	100
Monthly household income		
SAR 4,000–8,000	2	25
SAR 8,001–13,000	3	37.5
More than SAR 13,000	3	37.5
Educational level		
Diploma	1	12.5
Bachelor’s degree	6	75
Master’s degree or higher	1	12.5
Years of experience as a pediatric nurse		
1–2	2	25
3–4	2	25
5 or more	4	50

Nurses in this study faced many challenges in providing nursing care for children with ID. The following are the results of the thematic analysis. Initial codes were grouped into 12 subthemes. These sub-themes were then further abstracted into four higher-level themes.

### 3.2 Communication barriers and the need for caregivers/parents’ interpretation

#### 3.2.1 Lack of direct/verbal feedback and engagement

Communication challenges reported by nurses revolved around much uncertainty due to the lack of direct verbal feedback and engagement received by the patients with ID during assessments or providing nursing care and demanding on physiological changes and subtle signs.

The following cues were described by various participants when dealing with these children: “There was no contact with the patient, no eye contact, no body language.”

Some nurses expressed frustration about not being able to assess pain levels, potentially causing discomfort to the patient, and not getting immediate feedback to adjust care accordingly such as assessing the suitability of water temperature for a sponge bath or if the procedure is painful. This lack of verbal feedback leads to an emotional turmoil of feeling uncertain, inadequate, and guilty over not knowing whether they are meeting their patient’s needs or not:

“*If the child has a cannula I do not know if it’s painful for him when I flush, like I can’t take feedback from the patient [so] I don’t know what he is feeling.”*

“*In the morning care I don’t know if the water is too hot or too cold, [if it is] acceptable for the child or not*.”

Nurses may rely on physiological changes and subtle signs to try assess their patients’ needs because they lack traditional emotional cues and have varying levels of engagement, which make understanding their emotions even more complex. For instance, crying, elevated heart rate, or irritability are some of the cues nurses rely on to detect distress in their patients:

“*Sometimes we see tears coming out of their eyes or their heart rate fluctuate, we don’t see any facial expressions like normal kids*.”

The limited patient response may impact on the care delivered because nurses may focus on physical care and overlook the emotional needs that the children may have:

“*They can make you crazy. If they want your attention, they will disconnect themselves from the ventilator, desaturate, or hold their breath until you come*.”

#### 3.2.2 Challenges in interpreting patient behaviors

Sometimes nurses struggle to interpret their patients’ nonverbal behaviors and recognize their needs and suggest the need for specialized knowledge and training to understand these communication cues. They acknowledged that these cues have meaning; however, they found it difficult to decode them with their lack of expertise:

“*Sometimes they reflect themselves in the way we could not understand, maybe their action means something, but we are not the person qualified to understand these actions*.”

They reported that even the simplest of daily needs such as sleeping can be difficult to understand, which leads to frustration over delayed care for both patients and nurse. The child’s challenging behaviors could be an attempt to communicate a simple preference of need:

“*One patient was refusing to sleep because he [didn’t] like the color of the bedsheets … But it took us like 3 hours to understand*.”

Nursing care can be challenging, especially when caring for children with ID, who may resist because they do not understand what is happening. The following statements were reported by various participants:

“*This patient will need effort in dealing with him in preparing medication. I’ll take more time with the patient and in communicating with the patient*.”

“*The most difficult thing is miscommunication and irritability of some patients. Maybe patients with Down syndrome are at more risk because they cannot understand*.”

### 3.2.3 Dependency on family/caregivers for interpretation

Nurses expressed how heavily dependent they are on parents/caregivers to deal with the lack of direct verbal feedback and engagement by the patient, interpret their needs and understand their preferences. Daily nursing care was facilitated when nurses had helpful information such as patients’ favorite color, comfort items, and routines that only parents/caregivers would know. Parents/caregivers helping with communication helped nurses feel more confident providing nursing provision:

“*One night, a girl was tachycardic, sweating, crying. We did everything, and nothing helped. Then the nanny came and said, ‘Give her her teddy bear.’ That was the issue*.”

The absence of parents/caregivers may lead to nurses feeling uncertain because they do not fully understand the child’s needs, as reported by one of the participants:

“*I feel better when the parent is there so I can communicate with her*.”

### 3.3 Increased nursing demands due to complex care

#### 3.3.1 The need for more time

Caring for children with ID requires considerably more time to provide care for children with ID, shaping nurse-to-patient ratios and workload supervision. The following statements were reported by the nurses:

“*I can handle like one patient per shift with ID … because this patient will need effort in dealing with*.”

Another reported:

“*Not more than two, we can’t handle more than two. Feeding, bed bath, toileting/diaper. We need [to] focus, we can’t handle more*.”

#### 3.3.2 Safety and vigilance

Nurses reported the need to maintain constant vigilance due to the risky and challenging behavior of some of these children. These behaviors may threaten the child’s safety at times, such as self-disconnection from medical devices and treatment interruptions.

“*The most difficult thing that we are dealing with [is] … they cannot understand. They are still young. They are more likely to remove the IV line [or] remove the chest tube*.”

The acquisition of focused attention, observation, and monitoring adds to the complexity and demands of nursing care, which may cause exhaustion such as through constant bedside presence. The following was stated:

“*[We] need to keep the environment safe around them and they need to be under observation as similar to critical cases*.”

#### 3.3.3 Family-related challenges: denial, overprotection, lack of involvement

Disclosing information about a new diagnosis to families and supporting them in coping, especially in the intensive care units add to the complexity of the nursing care provided to their patients. One participant reported:

“*Some families are anxious, especially if their child had their first admission*.”

Another reported:

“*They are (family) in denial, and we don’t know how to tell them that their child is going to be disabled for the rest of their life*.”

Some nurses found parents and caregivers challenging, especially when they interfered in and impacted the nursing care provided to their children:

“*Some parents are over caring as I said, [saying] ‘don’t put the blood pressure cuff [on] this hand, you put [it on the leg’ or ‘don’t put the pulse oximetry [on] this finger, use that finger.’ It takes more time to care*.”

Not having the family around and/or their lack of involvement and capability issues was also noted as challenging for nurses:

“*One of the biggest challenges as a private hospital for children with special needs who are admitted is that there is no one with the child in the daily visit because they consider the hospital as a private care home*.”

#### 3.3.4 Individualized care requirements

Nurses emphasized how the different and unique nature of each patient with ID creates complex challenges, requiring more time, understanding, and flexibility in care delivery tailored to the needs and demands of the patient. The participants reported:

“*Each one of these patient[s] [has] a unique need; you have to discover what things … comfort them and then what things trigger them to [make it] easy to take care of them*.”

“*For example, children with autism need the same nurse to stay with them the whole care period*.”

### 3.4 Adaptation to the greater complexity of care

#### 3.4.1 Feelings and emotions

Some nurses were devastated and full of guilt while looking after children with ID. It was challenging for the nurses to witness the children go through painful procedures, they had difficulty in communicating with them, and they were unable to understand their feelings. The following were stated:

“*In the beginning I used to feel so sad that these children go through pain and they are so little*.”

“*Sometimes I feel guilty that I don’t understand the child especially if the mother is not present, all I can do is suction [and] change diaper. I feel better when the parent is there so I can communicate with her*.”

#### 3.4.2 Seeking solutions

Nurses searched for strategies to deal with the complexity of caring for children with ID. Some of the proposed solutions to lessen the burden on the nurses and allow better care for children with ID were: reduce nurse–patient ratio, use toys to model procedures for children, and allow children to select the color of the cannula:

“*They have something they attach to specifically like a toy*.”

Nurses also suggested that knowing the child better would ease the care for the child with ID, which can be emphasized by good communication and history taking from parents:

“*And for the older child I will recommend that when we are taking the history from family, we must ask these questions [such as] ‘what does your child like to eat?*’

Using distractors is another solution found by the nurses:

“*Distract the child using the mother or with the mother, using [the iPad] when we put [in] the cannula, but we use that even with normal children*.”

Discussing the experiences between nurses is another suggestion provided to overcome the complexity of taking care of a child with ID:

“*Sharing experiences between nurses in groups would be helpful. What helped one nurse could help another nurse*.”

#### 3.4.3 Equality in treatment and communication

Some nurses treat all children equally regardless of their disability. This applies to children in the same age group; the nurses met their needs and provided the same care for everyone who required it. The following was stated:

“*Some of the children with CP (cerebral palsy), yes they have (intellectual issue) but I deal with them like I deal with any other child in relation to care*.”

### 3.5 Need for support for both nurses and caregivers/parents

#### 3.5.1 Nurses’ need for support

Nurses identified their need for special training, courses, and workshops on caring for children with ID, which would contribute to supporting the nurses in providing better care to those children. The following statements were reported:

“*If they attended such workshops, initially this [would] improve the knowledge of the nurse…. The workshop will help, if the knowledge changes, then the skills will change*.”

“*We need more preparation in school on how to deal with the children with special needs*.”

#### 3.5.2 Parents’ need for support

Nurses indicated that parents of children with disabilities need various types of aids including psychosocial, educational, and emotional support. For instance, nurses emphasized the importance of engaging parents in childcare and providing them with educational and psychosocial support that in turn improves the child’s outcome. A holistic approach and multidisciplinary care are another family support needs identified by the nurses. The following statements were reported:

“*For the parents I recommend that they would be given educational sessions or programs*.”

“*They need special psychological, emotional, and physical care. Sometimes they are in denial*.”

## 4 Discussion

This study explored the lived experiences of nurses in caring for children who have ID, and it illuminated all the emotional, communicative, and practical complexities embedded deep in this specialized type of care. The findings, including communication struggles; emotional labor; heightened vigilance; and the need for individualized, family-centered approaches, underscore a pattern consistent with previous literature on pediatric ID nursing.

Deep communication barriers that nurses encountered were captured in the results of this study. Because some patients did not give verbal feedback, failed to express emotions well, and showed atypical behavior, basic care tasks such as pain assessment or hygiene support became emotionally difficult and ethically ambiguous. These themes are corroborated by Lewis et al. (18), who also found that nurses in Australian pediatric acute care settings experienced similar ambiguity, attributing it to nonverbal signals and complex caregiver dynamics. These nurses displayed distress or discomfort by way of physiological changes as well as behavioral disturbances (25, 26).

Guilt, uncertainty, and frustration were all obvious as the emotional toll of these ambiguities, particularly without parents, who often serve as interpreters of their child’s needs. This speaks to the critical role that caregivers play in pediatric ID settings, with Mimmo et al. (14) finding that parent–nurse partnerships improve care personalization and reduce provider stress. Similar feelings were reported by nurses in a westernized country, in which nurses felt guilty about their inability to communicate with the other healthcare team members to provide a holistic treatment plan (27).

The results of this study also reveal that children with ID require significantly more time and constant vigilance from nurses, especially when children disconnect from medical devices, showing high-risk behaviors. This is strongly supported by a study conducted in Turkey by Aydın et al. (25), who also reported nurses requiring sustained vigilance to prevent self-harm from risky behaviors and ensure patient safety. Also, Ong et al. (13) analyzed clinical incidents among children with ID and revealed unpredictable behaviors along with communication difficulties being frequently involved in safety events aligning with this. To prevent adverse events, lower nurse-to-patient ratios, heightened monitoring, and trauma-informed approaches are needed according to these nursing experiences.

Also, the results stress the importance of customized care. For children, understanding the routines, the comfort items, and the caregiver assignments is key. Mimmo et al. (14) likewise noted that positive care experiences emerged when nurses put in deliberate effort to know the child, build rapport, negotiate roles, and learn alongside families. These relationships function as protective factors with regards to miscommunication and emotional distress. Care plans that are tailored and strategies that are sensory sensitive are therefore needed.

The findings stress that nurses depend on caregivers to interpret children’s needs, and that increases both uncertainty and guilt when families are either absent or disengaged. Ong et al. (13) observed many safety concerns first identified by parents. This observation does further underscore the caregivers’ dual role as both a support and an advocate. Successful care hinges upon shared learning, trust, clear role negotiation, and partnership. This partnership was in fact a key theme within Mimmo et al.’s model (14). These perceptions show that families avoid or negate, making care hard, and they highlight that families must use structured psychosocial support.

Different perspectives on satisfaction and psychological wellbeing of caregivers can affect how families are able to communicate with health professionals and manage their children’s health conditions (28). The type of communication with families may also depend on their psychological state if they are too upset or if no psychosocial support mechanism is in place, causing uncertainty for the families and nurses. However, family participation as in the Alnahdi study allows for nurse-patient communication and coordination of care (28). This approach indicates a need for culturally tailored structured psychosocial support programs within a framework of parent-nurse collaboration and stresses the necessity of considering the psychosocial well-being of caregivers and health professionals (28). Moreover, the authors elaborate on the impact of Islamic and cultural beliefs on caregiving, such as tawakkul (trust in God), sabr (patience) and strict gender and kinship roles in Saudi Arabia (29). Caregiving is collective in Saudi Arabia, mothers act as caregivers while fathers are considered protectors and financial providers in the family (28, 29). These studies highlighted that culturally congruent, family-centered nursing practice must account for the sociocultural traditions and religious beliefs of Saudi Arabia, the roles of family members. Nurses, who are culturally sensitive, work with families, promote emotional health for caregivers, and maintain partnerships for the ensured safety and family-centered care of children who have intellectual disabilities.

The findings reveal that nurses struggle when dealing with children with ID. Nurses’ emotional status is affected when they are unable to understand the children’s feelings. Effective communication between the nurse and the patient is crucial to providing the patient with quality care. However, ID limits the communication between the care provider and the patient, especially if the patient is very young or non-verbal. Handling the children’s difficult behaviors and communicating with them were the most challenging issues nurses reported when dealing with children with ID in previous studies (30).

The nurses in our study highlighted multiple issues that limit their ability to maintain a high standard of care for patients with challenging conditions such as patients with ID. Some of the nurses stated that they have not been educated about how to manage children with ID. There is a lack of specialized training programs to provide a clear educational guide to supporting children with ID (31). The need to integrate specific courses on how to care for children with disabilities has been discussed in literature. The basic nursing program should outline a detailed manual to guide nurses and assist them in accommodating the physical as well as the psychological needs of the child. In a study conducted in Athens, the authors found that pediatric nurses had unfavorable attitudes toward children with disabilities that could be attributed to inadequacies in nursing education (31). The caregivers of children with ID face various emotional, physical, and even financial challenges. Health-care providers should support those parents and alleviate their burden. The nurses in our study emphasized the need to empower vulnerable parents. Parents of children with ID face an unbearable amount of psychological stress from caring for their children. Their distress could stem from any of the following issues: uncertainty about their children’s diagnosis, low socioeconomic status, feeling unprepared to support their children’s development, and lack of psychological support (32). In a study by Oulton et al. (17), parents of children with ID described how health-care professionals can collaborate with them to create effective partnerships that support the children’s health. The parents advised the health-care providers to prepare for children with ID being admitted to hospital, make it accessible for children with disabilities, provide the family with full and accurate information about the child’s needs, and respect the parents’ knowledge and ability (17). These recommendations show that multidisciplinary collaboration between all team members as well as the hospital administrators is vital to providing effective care.

One of the solutions suggested by the nurses to support the provision of quality care was to reduce the nurse–patient ratio. High ratio levels are associated with longer ICU stays and higher morbidity rates. Health-care administrators should consider not only the ratio but also the competencies of the staff (33, 34). In critical care units, the ratio should not exceed 1:2 to maintain quality care. Caring for children with ID requires extra time to understand the children’s individual needs. In addition, handling children with ID demands professional competencies and skills (18). Consistently with this, researchers in Iran also reported that shortage in staff and limitation in resources limit the nurse’s ability to provide high quality care (35).Therefore, nurses’ background, experience, training levels, and workload should be considered before assigning them to cases with ID.

### 4.1 Implications

This study provides several implications for clinical practice and research. Implications for clinical practice include tailored strategies for improving the care for children with ID that are based on the nurses’ experience. Further, effective communication methods should be used to support the interaction and understanding between the nurses and children with ID. Also, it is important to take into consideration the care for children with ID in determining the patient–nurse ratio to ensure the safety and quality of care provided. Given that the sample in this study consisted of female nurses, future research should include participants of all genders and from multiple settings to provide a more comprehensive understanding of the nursing experiences. Moreover, future researchers could use the findings from this study to develop interventional programs that support compassionate and comprehensive care for children with ID.

### 4.2 Limitation

The sample size in this study is relatively small, included only female nurses, and from limited settings, which may limit the transferability of the study. In addition, the social desirability bias may influence the perspectives of nurses in this study.

## 5 Conclusion

This study revealed that nursing care for children with ID is challenging and goes beyond routine pediatric care. Nurses described their experience and mentioned the major challenges including communication barriers, time constraints, and the need for presence of family. Although nurses described different ways of adapting to the complexity of the care, there is a critical need for support for nurses and families of children with ID. Providing resources, educational programs, or training sessions for both nurses and parents of children with ID is essential to ensure comprehensive care.

## Data Availability

The datasets presented in this article are not readily available to maintain confidentiality. Requests to access the datasets should be directed to saalghamdi6@kau.edu.
